# Supplementary material for: Drug repurposing for aging research using model organisms
Source: Aging Cell. 2017 Jun 16;16(5):1006–15. doi: 10.1111/acel.12626 (PMC5595691; doi:10.1111/acel.12626)
Supplement: Supplementary file 7 — Data S1 Zip‐Archive of all report cards. [file ACEL-16-1006-s007.zip › RC_GDS.pdf]

GDS

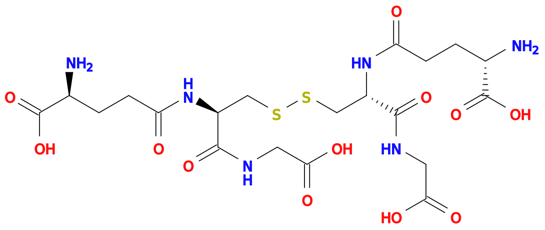

Database identifiers

ChEMBLCompound	CHEMBL1372
CHEBI	17858
eMolecules	482701

Ranking

	Rank	Score
Drosophila	417/697	0.343
C. elegans	390/591	0.147

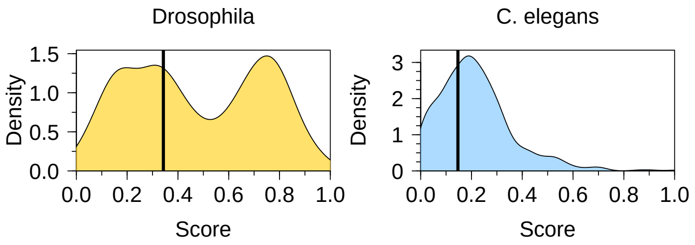

	Ageing implication	Domain conservation	Binding site conservation	Binding affinity	Bioavailability	Lipinski	Promiscuity	Purchasability	Drug approval	**Total**
Drosophila	1.0	0.782	0.606	0.688	(0.9)	-0.15	-0.0	0.1	0.1	0.343
C. elegans	1.0	0.933	0.727	0.688	0.208	-0.15	-0.0	0.1	0.1	0.147

Names

- **glutathione disulfide**
- GSSG
- oxidised glutathione
- Oxiglutatione

Roles

ChEBI entry 17858 has no roles Glutathione reductase

Status

Approved drug (according to ChEMBL)	Yes
Number of Rule of 5 violations	3
Binding affinity to original target in log units (RF-Score prediction)	5.79
Burns *C. elegans* bioavailability prediction	-6.34

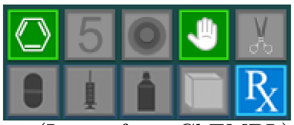

(Image from ChEMBL)

Compound Target Characteristics

Glutathione reductase, mitochondrial

Best gene implication in ageing for this target family came from gene P91938 annotated in UniProt release 2014_02. Annotation GO 8340 (determination of adult lifespan) was Inferred from Mutant Phenotype

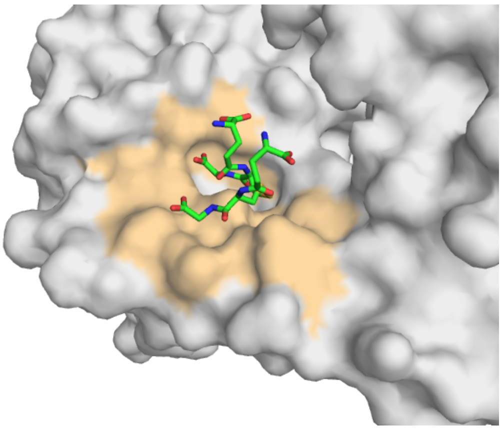

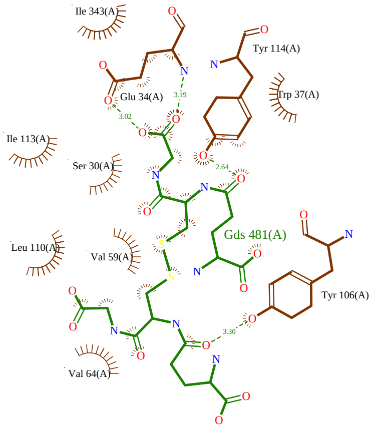

protein	amino acids contacts (binding site)
PDB:2grt:chainA:P00390	S E W V V Y L I Y N I
sp:P00390:GSHR_HUMAN	S A R V V Y L I Y N I
tr:F1LRE1:F1LRE1_RAT	- - - - - Y L I Y N I
sp:P47791:GSHR_MOUSE	S A R V V Y L I Y N I
sp:P91938:TRXR1_DROME	S A K V I H V V T D V
tr:Q86DB5:Q86DB5_CAEEL	S A R V V Y L L Y G I
tr:Q93379:Q93379_CAEEL	S A R V V Y L L Y G I
sp:P41921:GSHR_YEAST	S A R V V Y L I Y N I

	whole protein		domain-based		contact-based	
protein	ident	simil	ident	simil	ident	simil
PDB:2grt:chainA:P00390	1.0	1.0	0.99	1.0	1.0	1.0
sp:P00390:GSHR_HUMAN	1.0	1.0	1.0	1.0	0.82	0.81
tr:F1LRE1:F1LRE1_RAT	0.68	0.76	0.62	0.72	0.55	0.0
sp:P47791:GSHR_MOUSE	0.8	0.89	0.86	0.96	0.82	0.81
sp:P91938:TRXR1_DROME	0.28	0.64	0.36	0.73	0.18	0.61
tr:Q86DB5:Q86DB5_CAEEL	0.51	0.74	0.6	0.86	0.64	0.73
tr:Q93379:Q93379_CAEEL	0.5	0.73	0.6	0.86	0.64	0.73
sp:P41921:GSHR_YEAST	0.44	0.73	0.49	0.77	0.82	0.81

Trxr-1 (FBgn0020653) associated phenotypes

aging defective, developmental rate defective, lethal - all die before end of P-stage, lethal - all die before end of pupal stage, neuroanatomy defective, partially, partially lethal - majority die, some die during pharate adult stage, some die during pupal stage, stress response defective

(Information from FlyBase)

Trxr-1 (UniProt:P91938) annotation

Function: Thioredoxin system is a major player in glutathione metabolism, due to the demonstrated absence of a glutathione reductase. Functionally interacts with the Sod/Cat reactive oxidation species (ROS) defense system and thereby has a role in preadult development and life span. Lack of a glutathione reductase suggests antioxidant defense in Drosophila, and probably in related insects, differs fundamentally from that in other organisms. (PubMed:11158675, PubMed:11525742, PubMed:11796729).

Cofactor: FADNote=Binds 1 FAD per subunit.;

Biophysicochemical properties: Kinetic parameters: KM=6.5 uM for NADPH (at pH 7.4) (PubMed:11158675, PubMed:11782468, PubMed:11796729, PubMed:11877442, PubMed:17385893,

PubMed:18211101, PubMed:18991392); KM=1 uM for NADPH (at pH 7.4, 2 mM EDTA, 100 mM KPO(4)) (PubMed:11158675, PubMed:11782468, PubMed:11796729, PubMed:11877442, PubMed:17385893, PubMed:18211101, PubMed:18991392); KM=1 uM for NADPH (isoform B at pH 7.4, 2 mM EDTA, 100 mM KPO(4)) (PubMed:11158675, PubMed:11782468, PubMed:11796729, PubMed:11877442, PubMed:17385893, PubMed:18211101, PubMed:18991392); KM=7.0 uM for dhd (at pH 7.4, 200 uM NADPH, 100 mM KPO(4)) (PubMed:11158675, PubMed:11782468, PubMed:11796729, PubMed:11877442, PubMed:17385893, PubMed:18211101, PubMed:18991392); KM=141 uM for dhd (at pH 7.0, 0.15 mM NADPH, 1 mM EDTA, 1 mg/ml insulin, 50 mM KPO(4), 25 degrees Celsius) (PubMed:11158675, PubMed:11782468, PubMed:11796729, PubMed:11877442, PubMed:17385893, PubMed:18211101, PubMed:18991392); KM=7 uM for dhd (at pH 7.4, 2 mM EDTA, 100 mM KPO(4)) (PubMed:11158675, PubMed:11782468, PubMed:11796729, PubMed:11877442, PubMed:17385893, PubMed:18211101, PubMed:18991392); KM=19 uM for dhd (isoform B at pH 7.4, 2 mM EDTA, 100 mM KPO(4)) (PubMed:11158675, PubMed:11782468, PubMed:11796729, PubMed:11877442, PubMed:17385893, PubMed:18211101, PubMed:18991392); KM=5.9 uM for Trx-2 (at pH 7.4, 100 uM NADPH, 2 mM EDTA, 100 mM KPO(4)) (PubMed:11158675, PubMed:11782468, PubMed:11796729, PubMed:11877442, PubMed:17385893, PubMed:18211101, PubMed:18991392); KM=310 uM for 5,5'-dithiobis-2-nitrobenzoic acid (DTNB) (at pH 7.4, 100 uM NADPH, 100 mM KPO(4)) (PubMed:11158675, PubMed:11782468, PubMed:11796729, PubMed:11877442, PubMed:17385893, PubMed:18211101, PubMed:18991392); KM=0.17 mM for DTNB (at pH 7.0, 0.2 mM NADPH, 10 mM EDTA, 100 mM KPO(4), 25 degrees Celsius) (PubMed:11158675, PubMed:11782468, PubMed:11796729, PubMed:11877442, PubMed:17385893, PubMed:18211101, PubMed:18991392); KM=380 uM for DTNB (at pH 7.4, 2 mM EDTA, 100 mM KPO(4)) (PubMed:11158675, PubMed:11782468, PubMed:11796729, PubMed:11877442, PubMed:17385893, PubMed:18211101, PubMed:18991392); KM=410 uM for DTNB (isoform B at pH 7.4, 2 mM EDTA, 100 mM KPO(4)) (PubMed:11158675, PubMed:11782468, PubMed:11796729, PubMed:11877442, PubMed:17385893, PubMed:18211101, PubMed:18991392); KM=675 uM for methylseleninate (100 uM NADPH) (PubMed:11158675, PubMed:11782468, PubMed:11796729, PubMed:11877442, PubMed:17385893, PubMed:18211101, PubMed:18991392); Vmax=24.3 umol/min/mg enzyme toward NADPH (at pH 7.4) (PubMed:11158675, PubMed:11782468, PubMed:11796729, PubMed:11877442, PubMed:17385893, PubMed:18211101, PubMed:18991392); Vmax=16 umol/min/mg enzyme toward Trx-2 (at pH 7.4, 100 uM NADPH, 2 mM EDTA, 100 mM KPO(4), 25 degrees Celsius) (PubMed:11158675, PubMed:11782468, PubMed:11796729, PubMed:11877442, PubMed:17385893, PubMed:18211101, PubMed:18991392); Note=Measurements were conducted with isoform A unless noted otherwise.; pH dependence: Optimum pH is 7.6 for isoform A with Trx-2 and NADPH as substrates. (PubMed:11158675, PubMed:11782468, PubMed:11796729, PubMed:11877442, PubMed:17385893, PubMed:18211101, PubMed:18991392);
Subunit: Homodimer. (PubMed:17385893).
Subcellular location: Isoform B: Mitochondrion.
Subcellular location: Isoform A: Cytoplasm.
Tissue specificity: During embryogenesis, expression is seen in germ cell progenitors, developing midgut, hindgut and proventriculus. (PubMed:11525742).
Developmental stage: Expressed both maternally and zygotically during all stages of development, highest expression during adult stages. (PubMed:11525742, PubMed:11796729).
Miscellaneous: The active site is a redox-active disulfide bond.
Caution: Was originally thought to be a glutathione reductase. (PubMed:9056265).

(Information from UniProt)

gsr-1 (WBGene00008117) associated phenotypes
lethal, sterile

(Information from WormBase)
